# Active cycle of breathing technique versus oscillating PEP therapy versus walking with huffing during an acute exacerbation of bronchiectasis: a randomised, controlled trial protocol

**DOI:** 10.1186/s12890-023-02324-8

**Published:** 2023-01-25

**Authors:** Jennifer Phillips, Wayne Hing, Rodney Pope, Ashleigh Canov, Nicole Harley, Annemarie L. Lee

**Affiliations:** 1 Physiotherapy, Faculty of Health Sciences and Medicine, Bond University, 2 Promethean Way, Robina, QLD 4226 Australia; 2 Allied Health Department, The Wesley Hospital, Uniting Care Health, 451 Coronation Drive, Auchenflower, QLD 4066 Australia; 3 School of Allied Health, Exercise and Sports Sciences, Charles Sturt University, PO Box 789, Albury, NSW 2640 Australia; 4 Allied Health Department, St Andrews Hospital, Uniting Care Health, 457 Wickham Terrace, Spring Hill, QLD 4000 Australia; 5 Department of Physiotherapy, Faculty of Medicine, Nursing and Health Sciences, Monash University, Moorooduc Hwy, Frankston, VIC 3199 Australia; 6 Institute for Breathing and Sleep, 145 Studley Road, Heidelberg, VIC Australia

**Keywords:** Bronchiectasis, Airway clearance, Sputum clearance, Exacerbation

## Abstract

**Background:**

Airway clearance techniques (ACTs) for individuals with bronchiectasis are routinely prescribed in clinical practice and recommended by international guidelines, especially during an acute exacerbation. However, there is limited evidence of the efficacy of these techniques during an exacerbation to improve sputum expectoration, health-related quality-of-life (HRQOL) or exercise tolerance. The primary aim of this study is to compare the effects of the active cycle of breathing technique (ACBT), oscillating positive expiratory pressure (O-PEP) therapy, and walking with huffing on sputum expectoration for adults hospitalised with an acute exacerbation of bronchiectasis. Secondary aims are to compare the effects of these interventions on HRQOL, health status, exacerbation rates and hospital admissions in a six-month period following hospital discharge.

**Methods:**

This multi-centre randomised controlled trial will recruit adults with an acute exacerbation of bronchiectasis requiring hospital admission. Participants will be randomised to receive one of three interventions: ACBT, O-PEP therapy, and walking with huffing. Outcome measures including sputum volume during and 1-h post ACT session, and 24-h sputum, as well as health status, HRQOL and exercise capacity will be completed during inpatient stay on day 2 and day 6 of admission, and within 24 h of hospital discharge. Time to first exacerbation, and time to first hospitalisation will be monitored via monthly phone calls for six months post hospital discharge. Health status and HRQOL will be assessed after discharge at two and six months, and exercise capacity will be assessed at six months post hospital discharge.

**Discussion:**

Despite recommendations regarding the importance of ACT for individuals with bronchiectasis during an acute exacerbation, there is a gap in the literature regarding effectiveness of ACT when undertaken by individuals in this clinical state. This study will add to the evidence base regarding the effectiveness of commonly implemented ACTs during a hospital admission with an exacerbation of bronchiectasis. Additionally, it will contribute to knowledge of the long term effects on important and patient-centred outcomes, including incidence of future exacerbations, and HRQOL, which has not been previously established.

*Trial registration* Registered on the Australian and New Zealand Clinical Trials Registry (ACTRN12621000428864).

## Introduction

Bronchiectasis is a chronic and progressive respiratory condition which is characterised by chronic cough, sputum production, shortness of breath, recurrent exacerbations, and decreased exercise tolerance [1–3]. Recurrent exacerbations have been shown to lead to progressive deterioration of lung function [4] and poorer health-related quality of life (HRQOL) [5], and account for a higher mortality rate [6]. An exacerbation is defined as a deterioration in three or more of the following symptoms for at least forty-eight hours: cough; sputum volume and/or consistency; sputum purulence; haemoptysis, breathlessness and/or exercise tolerance; fatigue and/or malaise [7].

Airway clearance techniques (ACTs) are routinely used as part of a physiotherapy treatment regimen for people with bronchiectasis, with most individuals experiencing excessive secretions due to increased sputum production and difficulty in clearing these secretions secondary to airway damage [2, 8–10]. The goals of ACTs are to provide more effective sputum clearance to improve ventilation, as well as reduce cough and breathlessness impact, therefore improving HRQOL [11]. International guidelines recommend ACTs are taught routinely to people with bronchiectasis, and state that during an acute exacerbation of bronchiectasis, an individual should be reviewed daily and that a change in ACT may be required [12–14]. Previous research on ACTs has been completed on individuals who are clinically stable [15–17], but there is little evidence available regarding the effectiveness of ACTs during an acute exacerbation [10, 15]. Furthermore, due to differences in sputum volume and consistency, extrapolating evidence from those in a stable clinical state may be misleading in estimating the effect for individuals during an acute exacerbation [18].

Two of the most commonly implemented ACTs internationally are the active cycle of breathing technique (ACBT) and oscillating positive expiratory pressure (O-PEP) therapy [8, 19–23]. In a recent survey of physiotherapists working in Australia and New Zealand, both the ACBT and O-PEP therapy were found to be frequently used [23]. The ACBT was used very often or always by 89% of participants managing adults with an acute exacerbation of bronchiectasis [23]. It was perceived to be effective in this patient population by 90% of physiotherapist participants in the same study. The same survey reported that O-PEP therapy was used routinely by 75% of participants and was perceived to be very effective or effective by 97% of clinician participants managing adult patients during an acute exacerbation of bronchiectasis [23]. In a recent study investigating ACT use in individuals with bronchiectasis in the United States by analysing data from the United States Bronchiectasis Registry, the most commonly used ACT was O-PEP devices (44%) followed by multiple modalities (43%) [21]. A study exploring ACT use based on findings from the European Bronchiectasis Registry (EMBARC) found the most commonly implemented ACT was the ACBT (26.8%) [22].

The ACBT is an ACT which uses modulation of breathing to employ the physiological effects of interdependence and collateral ventilation to improve ventilation, which when combined with a huff will aid secretion clearance [11]. The ACBT has been used for many years in a range of respiratory conditions [24], requires no equipment and can be completed in differing positions [11, 25].

Oscillating PEP therapy involves a device designed to provide positive expiratory pressure and endobronchial pressure oscillation to loosen secretions from the airway walls [26, 27]. There are a range of devices available including Aerobika®, Flutter Valve®, Acapella® and Lung Flute® [21]. All devices are designed to utilise collateral ventilation to improve ventilation distribution, and direct airflow behind secretions, as well as splint open the airways using positive expiratory pressure [28]. The oscillation of endobronchial pressure is designed to alter the viscosity of secretions to allow their movement towards the larger airways in preparation for expectoration [28].

The goal of ACBT and O-PEP therapy is to facilitate secretion removal, therefore an improvement in sputum removal would correspond with an increase in sputum expectorated during or immediately following an airway clearance session, plus or minus a decrease in sputum expectoration in the 24 h following an airway clearance session [17, 29, 30]. A recent international guideline also recommended implementation of the ACBT or O-PEP therapy for individuals with stable bronchiectasis (level D—low level evidence), based on current evidence available [12]. In individuals with a range of chronic respiratory diseases, the ACBT has been shown to be beneficial in the short term for improving sputum wet weight, forced expiratory volume in 1 s (FEV_1_) and forced vital capacity (FVC), when compared to other types of ACTs [24, 31]. Oscillating PEP therapy has been shown to be equal to other ACTs in improving sputum expectoration and lung function [15], and demonstrated improvements in both sputum expectoration and HRQOL in individuals with stable bronchiectasis compared to no treatment [16, 28]. It has also been shown to be a preferred ACT by individuals with bronchiectasis compared to other ACT options, which may improve compliance with therapy [10].

Although both the ACBT and O-PEP therapy have demonstrated benefits in patients who are clinically stable, [25, 28, 32, 33], there is limited evidence for these techniques in individuals experiencing an acute exacerbation of bronchiectasis. One systematic review investigated ACTs during an acute exacerbation and highlighted the limited evidence available, with only six studies published, and encompassing 120 participants [10]. The key findings were all implemented ACTs appeared safe during an acute exacerbation and the ACBT was found to be superior in sputum expectoration (greater decrease in 24 h collection), cough-related quality of life and gas exchange when compared to more traditional ACTs of percussion and postural drainage [10]. Oscillating PEP therapy was preferred by participants over other types of ACTs [10]. However, the long-term effects of these treatments on clinically important and patient-centred outcomes, including incidence of future exacerbations, HRQOL and health status have not been established [10].

Exercise or physical activity is routinely used as part of physiotherapy management of individuals with bronchiectasis, including during an acute exacerbation [19, 34] and has recently been reported as a potential substitute for traditional ACTs in patients with chronic respiratory diseases [35]. Traditionally, walking is the most commonly clinically implemented exercise during an acute exacerbation, and the goal is to maintain function, minimise the effect of bed rest and facilitate hygiene care and activities of daily living [36]. The potential for it to also be a standalone ACT is based on the physiological rationale that exercise, particularly walking, may increase expiratory airflow, decrease sputum mechanical impedance, improve ease of expectoration, increase volume of secretions cleared and increase cough [37, 38]. Although exercise as a standalone ACT has not been investigated in bronchiectasis, there is emerging evidence for this option in cystic fibrosis (CF) and chronic obstructive pulmonary disease (COPD) [38, 39]. Exercise as an ACT in the cystic fibrosis population has been shown to improve ease of expectoration and sputum clearance when compared to rest, and may have similar short-term effects for airway clearance as traditional ACTs [35, 36]. Exercise has also been explored in individuals with COPD, with one study investigating the effect of usual care including an exercise program compared to PEP therapy during an acute exacerbation of COPD [39]. The exercise program consisted of implementing a walking program (or equivalent lower limb exercise), with the aim of achieving 30 min/day combined with coughing. No significant differences were found between groups on symptoms, quality of life and future exacerbations, suggesting usual care incorporating exercise and coughing is just as effective as PEP therapy in achieving key outcomes following acute exacerbations of COPD [39]. Although, traditionally, evidence for ACT and other therapies for bronchiectasis has been extrapolated from other respiratory populations, there are some key differences between these conditions which highlight the importance of research for bronchiectasis. Of particular relevance are the differences in sputum rheology and electrolyte content [40]. The differences in sputum characteristics may lead to ACTs demonstrating differing results in clinically important outcome measures.

### Research aims

The primary aim of this randomised controlled trial is to compare the effects of the ACBT, O-PEP therapy and walking with huffing (control condition) on sputum expectoration for adults hospitalised with an acute exacerbation of bronchiectasis. Secondary aims are to compare the effects of these three interventions on HRQOL, health status, exacerbation rates and hospital admissions in a six-month period following this hospital admission.

## Methods

### Design

This study will be a 6-month prospective, parallel-groups, randomized, controlled trial comparing the short and long-term effects of ACBT, O-PEP therapy, and walking with FET, in adults hospitalised with an acute exacerbation of bronchiectasis. The full study protocol was registered with the Australian and New Zealand Clinical Trials Registry (ACTRN12621000428864). Ethics approval was received from the Uniting Care Human Research Ethics Committee (Reference number 2020), Bond University Human Research Ethics Committee (Reference number UCH HREC2020), Charles Sturt University Human Research Ethics Committee (Protocol number H21027) and Monash University Human Research Ethics Committee (Project number 27817).

### Participants

Participants will be adults (age 18 years and over) admitted to one of two collaborating private hospitals in Brisbane, Australia, with an acute exacerbation of bronchiectasis, defined by internationally accepted criteria [7]. Exclusion criteria include: primary diagnosis of a respiratory condition other than bronchiectasis, breathing through an artificial airway or requiring non-invasive ventilation within 48 h of hospital admission, or any contraindication to ACBT or O-PEP therapy, including undrained or drained pneumothorax within the last 6 months, post lung lobectomy or lung transplant, haemodynamic instability or severe cardiovascular disease, undrained empyema or lung abscess, active haemoptysis, middle ear infection, previous recruitment and completion/withdrawal from this study, or hyper-reactive airways as diagnosed by a respiratory physician during the hospital admission [41–43]. Potential participants requiring high flow oxygen therapy will still be eligible to participate. A recent hospital admission/exacerbation will not exclude potential participants from being included in the study.

Participants will be assessed for potential inclusion in the first 48 h of hospital admission; if they meet any of the exclusion criteria at this time, they will be deemed ineligible and not reassessed for inclusion later in their hospital admission.

### Recruitment and randomisation

**Fig. 1:**
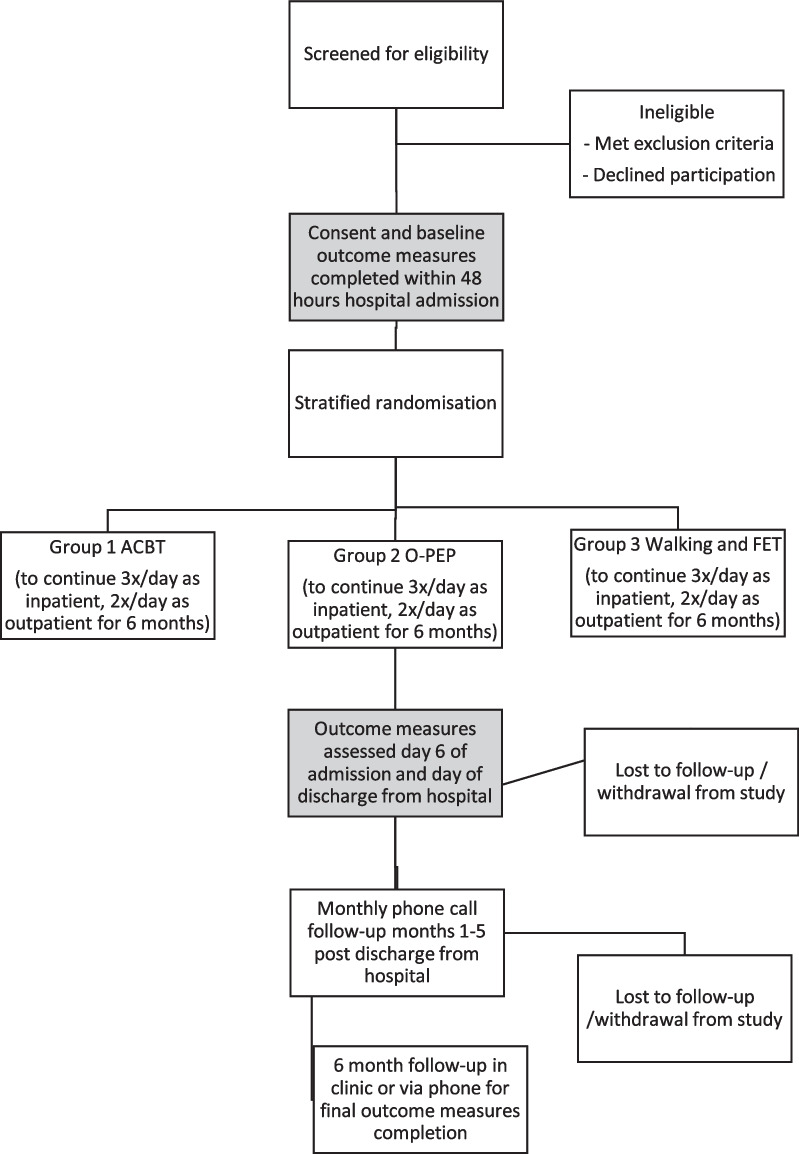
Participant flow diagram. *ACBT* active cycle of breathing technique, *FET* forced expiration technique, *O-PEP* oscillating positive expiratory pressure

**Fig. 2:**
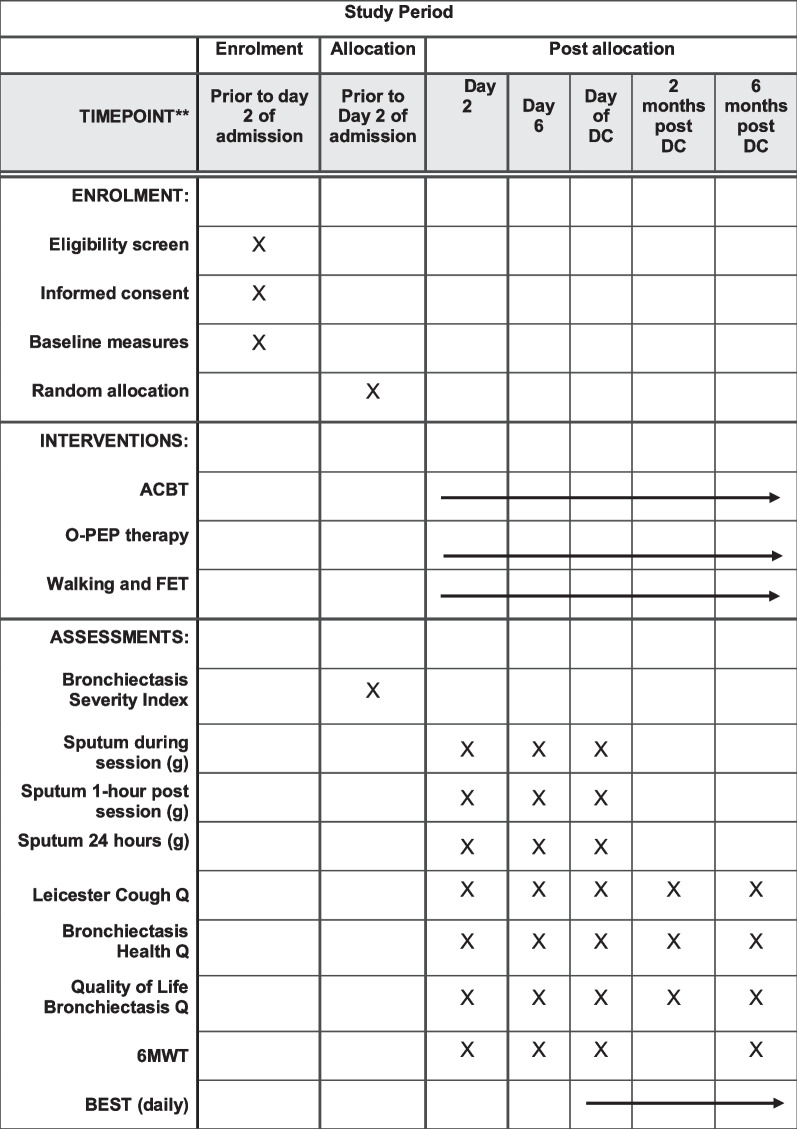
Participant timeline. *ACBT* Active cycle of breathing technique, *BEST* Bronchiectasis Exacerbations and Symptoms Tool, *DC* Discharge, *g* Grams, *O-PEP* Oscillating positive expiratory pressure therapy, *Q* Questionnaire, *6MWT* Six-minute walk test

The flow of participants through the study is available in Fig. 1. The participant timeline is available in Fig. 2. Potential participants will be identified via two means; firstly, the research assistant checking for new hospital admissions daily, secondly; new admissions will be flagged to the research team from nursing, medical, and physiotherapy staff who identify potential participants. Screening for potential participants will occur seven days per week during the participant recruitment phase. Additionally, training sessions for physiotherapy staff at both hospitals will be run by the chief investigator throughout the recruitment period to ensure all staff are aware of the study and inclusion criteria. Once identified, potential participants will initially be approached by the chief investigator or a research assistant to provide verbal and written information on the study protocol. If the potential participant provides written consent to participate in the study, then baseline outcome measures will be collected prior to group allocation.

Baseline assessment of the severity of bronchiectasis will be completed on enrolment to the study, and prior to random allocation to intervention groups, and scored according to the Bronchiectasis Severity Index, a validated measure of disease severity [6]. During the hospital admission, growth of bacterial organisms (such as Pseudomonas Aeruginosa, Haemophilus Influenzae) in sputum samples will be recorded [14] for further characterisation of specific presentations of participants.

### Allocation

Participants will be randomly assigned to one of three groups, based on a computer-generated random sequence, with the allocation stratified by participant self-reported usual volume of sputum production (low volume (< 1 teaspoon sputum per day when well) or high volume (> 1 teaspoon sputum per day when well) [44]) to ensure a balance between groups in this characteristic. The computer-generated random allocations will be placed into numbered sequences (one for each strata) of opaque envelopes by the chief investigator, which will only be opened by the treating physiotherapist once baseline measures are completed. Only participants and treating physiotherapists will have knowledge of the group allocation. Blinding of assessors to treatment allocation will be achieved through the assessor not accessing medical documentation for the participant, participants being requested to place any handouts or equipment out of sight (e.g. in a drawer) prior to the assessor entering the room, and the participants being asked not to discuss any intervention with the assessor. The assessors will report any instances of unblinding and these will be recorded. The assessors will be senior cardiorespiratory physiotherapists who are not involved in the patient’s care during the admission.

### Outcomes

All participants will be assessed at three time points during their hospital admission, and then once per month for six months post hospital discharge, by an assessor blinded to group allocation. The assessments to be conducted are described below. If participants withdraw from the study prior to completing the six months follow-up, their data will be included in data analysis up until the point of withdrawal unless specially requested otherwise by the participant.

The primary outcome of sputum volume, measured by the wet weight of sputum (grams), will be assessed three times during hospital admission on day 2, day 6, and within 24 h of hospital discharge. On each occasion, sputum will be collected during a physiotherapist-supervised ACT session, in the hour following the ACT treatment session, and over a 24-h period following the session. Twenty-four-hour sputum volume has been shown to be a responsive outcome measure during acute exacerbations of bronchiectasis [29, 43, 45]. Either an increase in sputum volume during the session, or one-hour post-session, or a decrease in sputum volume in the following 24-h period will be interpreted as an improvement in sputum clearance [29, 30, 43].

Secondary outcomes to be assessed include health status (Bronchiectasis Health Questionnaire) [46], disease-specific HRQOL (Quality of Life-Bronchiectasis questionnaire (QoL-B) and Leicester Cough Questionnaire) [47, 48] and exercise capacity (6 min walk test [49]). Each of these measures will be completed three times during hospital admission (on day 2, day 6, and within 24 h of hospital discharge). Additionally, health status and HRQOL will be assessed at two- and six-months post hospital discharge. Exercise capacity will be completed at six months post hospital discharge. The questionnaires will be completed via pen and paper during hospital admission, then via phone at two months post hospital discharge. At six months the questionnaires will be completed via paper and pen in the clinic or via phone if participants are unable to attend in person. The questionnaires for health status and HRQOL have been shown to be valid and reliable measures in individuals with bronchiectasis [46–48, 50].

Number of exacerbations, number of hospital admissions and time to first exacerbation and hospital admission will be collected for six months post hospital discharge from the date of discharge. A blinded assessor will complete monthly phone calls using questions investigating clinical status in the last month including symptoms, hospital admissions and use of antibiotic therapy (and comparing participant responses to these questions to the international definition of an exacerbation for bronchiectasis [7]. Participants will also complete the Bronchiectasis Exacerbations and Symptoms Tool (BEST) daily from time of discharge from hospital to six month follow-up [51]. The BEST tool is a symptom diary, designed to detect bronchiectasis exacerbations, which involves six questions regarding breathlessness, sputum volume, cough, fatigue, sputum colour and cold and flu symptoms. It has been shown to be responsive to identifying exacerbations at onset and recovery for individuals with bronchiectasis [51].

### Interventions

Participants will be randomly allocated to one of three groups, which are based on the intervention to be received:

1. The ACBT
2. O-PEP therapy
3. Walking and FET (control)

The instructions for use of each ACT will be informed by practice guidelines [52] and previous studies [15, 23]. Whilst inpatients, participants will be instructed to complete their allocated ACT three times daily, once supervised by a physiotherapist, and twice independently. On discharge, participants will be instructed to continue their allocated technique twice daily. Participants will be instructed to cease their usual airway clearance therapy (if any) and exclusively complete the allocated technique for the duration of the study period. Compliance will be encouraged and monitored through daily interviews (inpatient stay), provision of written instructions, and monthly phone calls (upon discharge). The phone calls will collect information regarding adherence to ACT and clinical status in the previous month, including changes in respiratory symptoms, any hospital admissions or use of antibiotics.

#### Usual care

Participants in all three groups will receive usual care in addition to the allocated airway clearance intervention. Usual care will consist of medical therapy, prescribed by the patient’s treating physician (including antibiotics, corticosteroids, supplemental oxygen with or without humidification, nebulised medication), nursing care, and other allied health assessment and intervention as required. No other ACTs will be delivered as part of usual care. In the event of an emergency or significant clinical deterioration, the ward physiotherapist may treat the participant as they deem necessary, and the participant will then be returned to the allocated intervention when safe to do so. Any such instances will be recorded and reported. If a participant was treated with an alternative ACT for more than 48 h during their hospital admission, they will be excluded from secondary per-protocol analyses of the trial but included in the primary intention-to-treat analyses.

As part of usual care, all participants will receive a physical exercise training regimen based on ground-based walking, prescribed by the ward physiotherapist with the aim of returning to previous levels of function throughout admission [36, 39]. During this activity, participants will be monitored with the goal to maintain clinically acceptable, individually determined blood oxygen saturation levels and heart rate range parameters.

#### Group 1: the ACBT

Participants will complete the ACBT according to guidelines [24, 52], positioned sitting upright in a chair (or sitting up in bed if unable to sit in a chair). The ACBT consists of three components: breathing control, thoracic expansion exercises and forced expiratory technique (FET). The cycle implemented will be:

1. Breathing control—participant to breathe at their usual rate and depth
2. Thoracic expansion—participant to breathe in slowly and deeply, pause for 1–2 s, breathe out fully but not forcefully. Repeated 2 to 3 times.
3. Return to breathing control as above
4. Thoracic expansion exercises as above repeated 2–3 times.
5. Return to breathing control as above
6. FET—participant to take a slightly deeper breath than normal in, open mouth and keep it ‘O’ shaped, then breathe out more forcefully using abdominal muscles to assist. This breath out should sound like a forced sigh. Participants to repeat 2 to 3 times, with breathing control in between as needed.
7. Return to breathing control until ready to begin another cycle.

The participant may cough if needed throughout the cycle and will be instructed to complete a minimum of three cycles each session, or until the end points listed below. Participants will be advised to complete the ACBT 3 times per day whilst in hospital, and then twice daily on hospital discharge for the full six months of the study duration.

#### Group 2: O-PEP therapy

The O-PEP therapy group will use an Aerobika® device. Participants will be instructed to complete O-PEP therapy according to guidelines [52], positioned in forward lean sitting with elbows supported on a table and feet flat on the ground (or upright in bed if unable to sit in a chair). The participants will be instructed to complete 10 breaths, followed by a huff, and then a cough only if needed, a minimum of 3 times per session, or until the end points listed below. Participants will be advised to use the O-PEP device 3 times per day whilst in hospital, and then twice daily on hospital discharge for the full six months of the study duration.

#### Group 3: Walking and FET (control condition)

Participants randomly assigned to group 3 will complete a standardised exercise program based on ground-based walking in combination with directed FET. Participants will self-select their walking speed to a modified Borg scale level of 3–5 whilst maintaining clinically acceptable, individually determined blood oxygen saturation levels and heart rate range parameters [53]. The goal will be to complete a total of 30 min/day; while they are inpatients, participants will be instructed to walk for 10 min, 3 times per day combined with a series of huffs at the end of each session. As outpatients, participants will be instructed to maintain the goal of 30 min/day in combination with FET. The FET involves a huff in combination with breathing control. A huff involves breathing out with mild-moderate force to aid clearance of sputum from the lungs [25]. The following instructions will be provided to participants:

- Huff—participant to take a slightly deeper breath than normal in, open mouth and keep it ‘O’ shaped, then breathe out more forcefully using abdominal muscles to assist. It should sound like a forced sigh. Participants to repeat 2 to 3 times with breathing control in between as needed.

Participants may cough as required during or following the intervention.

#### End points

The end points for the treatment sessions in all three groups will be:

1. Two non-productive cycles plus clear huff
2. Terminated by participant or physiotherapist for other reasons (such as participant fatigue, or the participant reporting subjectively feeling clear).
3. Productive cough remaining, or huff not clear, but session reached maximum time of 30 min.

### Sample size

Sample size calculations were completed using G*Power software (version 3.1.9.6) to ensure the study was adequately powered. Based on previously published research, the minimal clinically important difference in sputum volume is 15 ml over 24 h [29]. To detect a difference at least as large as the minimal clinically important mean difference between groups, assuming a SD of 15 ml [29], power of 0.80 and alpha value of 0.05, 17 participants per group were required, giving a total of 51 participants required for the study. To accommodate a 40% attrition rate, 90 participants will be sought [29].

Due to the impact of COVID-19, rates of bronchiectasis exacerbations have reduced significantly in response to lock-down measures and the use of masks and social distancing in the community [54]. This reduction in exacerbation rates, and therefore hospitalisation rates, may impact rate of participant recruitment [54]. To minimise this impact, the time frame for participant recruitment will be continued as long as resources allow to maximise participant numbers. The sample size calculated allows for substantial attrition, and the authors acknowledge this may not be possible within resources constraints.

#### Statistical methods

Data analysis will be performed using SPSS V.28 (SPSS, Chicago, Illinois, USA). Data will be analysed using intention-to-treat principles, with alpha set at 0.05 for inferential analyses.

Data will be described as mean (standard deviation) or median (interquartile range) for continuous outcomes depending on distribution, and as frequency (percentage) for categorical outcomes. Demographic data, including age, pseudomonas aeruginosa colonisation, bronchiectasis severity scores and QoL-B scores will be compared to findings from the Australian Bronchiectasis Registry to determine external validity of the study findings. Where participants have a comorbid respiratory condition (such as Asthma or COPD), this will be noted and reported in the data analysis, and considered during interpretation of the findings of the study and when discussing the generalisability of findings.

The primary outcome, between-group difference in sputum volume over a 24-h period will be assessed using mixed-effects linear regression models, with treatment group and time included as main effects, and a group-by-time interaction term. Participant will be included as a random effect to account for the repeated measures nature of the data.

Secondary outcome measures with continuous outcomes will be analysed using linear regression, with binary outcomes using logistic regression, and with count outcomes using Poisson regression models. Repeated measures models will be used when appropriate. When data does not fit regression assumptions, non-parametric methods such as the Kruskal–Wallis test will be used to compare differences in scores on each instrument between groups at each of the different time points. Time-to-event outcomes, such as time to first recurrence of an exacerbation following discharge from initial hospital admission, will be visualised using Kaplan Meier survival curves and analysed using Cox proportional hazards regression.

#### Ethical considerations

The chief investigator or research assistant will approach potential participants, and provide the information sheet and informed consent form, as well as address any questions or concerns potential participants have. Participants will be informed in writing that their participation in the research study is voluntary, and that if they decide to participate, they can withdraw at any time without providing a reason. Participants will be advised that if they decide not to participate or withdraw from the research study it will not disadvantage them in any way. They will also be advised that their decision to participate or not will not affect their access to medical services or their relationship with any health care professionals involved in their care. Potential participants will have up to one day to consider participation prior to enrolment. The treating clinical staff will not initially approach potential participants about the study, to minimise the risk of coercion. All data will be de-identified through the use of assigned unique codes.

#### Monitoring

Data monitoring will be performed by the chief investigator throughout the data collection process, with the support of the wider research team, to ensure data quality is maintained.

The researchers will monitor the conduct and progress of the research through regular team meetings for the project duration, and annual reports to the ethics committees. The research assistant and the treating ward physiotherapists in collaboration with the chief investigator will monitor the participants throughout the study and advise the participants’ treating respiratory physicians if a significant deterioration in any outcome measure is detected. Any adverse events will be recorded/documented by the treating physiotherapist or research assistant and reported to the chief investigator, whether the event is related to the research study or not. If any serious adverse events occur that are related to the study during the data collection time, these will be reported promptly to the chief investigator of the study, who will then urgently report the event to the relevant ethics committees. Any reported events will be reviewed in detail by the research team to ensure that the event does not occur again.

The study will end once all data has been collected or upon completion of funding/ethical approval. No interim analyses are planned. There are no provisions available for post-trial care, however education will be provided by a physiotherapist regarding continuing an appropriate airway clearance regimen at their final review as part of the study.

#### Dissemination

Once data collection has been completed, the authors intend to publish the findings in a peer reviewed journal. This project forms part of the chief investigator’s higher degree by research. Once published, results of the trial will be shared with all participants who consented and chose “yes” when prompted regarding if they would like to hear the results of the study upon completion.

## Discussion

The details of the background and methodology for a randomised clinical trial designed to investigate the effectiveness of ACTs during an acute exacerbation of bronchiectasis have been outlined. This field of enquiry has been identified as a research priority by the British Thoracic Society [12]. The ACTs to be investigated are the ACBT and O-PEP therapy with a third group (control group) walking with FET. These ACTs are commonly implemented therapies internationally for individuals with bronchiectasis [21–23]. The trial proposed, to the authors knowledge, will be the first sufficiently powered for the primary outcome measure of sputum volume in 24 h in this patient population. Additionally, the six month follow up will be one of the longest investigating ACTs for individuals with bronchiectasis [10].

Bronchiectasis is a chronic respiratory lung disease characterised by a chronic cough, breathlessness, fatigue and recurrent exacerbations that have an impact on HRQOL [2, 12, 55]. Airway clearance techniques are routinely prescribed in clinical practice, and recommended by international guidelines for individuals with bronchiectasis, particularly during an exacerbation [12, 13, 21, 23]. Despite this, there is very limited evidence investigating which ACT should be implemented (10).

Due to current lack of guidance for clinical practice, the results from this study will inform physiotherapy practice in caring for individuals with an acute exacerbation of bronchiectasis. The long-term follow-up will also provide useful information regarding prescription of ACTs post hospital discharge for individuals with bronchiectasis.

## Data Availability

As this is a study protocol, there is no data available. Materials may be available via written request to the chief investigator.

## Abbreviations

ACBT: Active cycle of breathing technique
ACT: Airway clearance technique
BEST: Bronchiectasis exacerbation symptom tool
CF: Cystic fibrosis
COPD: Chronic obstructive pulmonary disease
EMBARC: European Multicentre Bronchiectasis Audit and Research Collaboration
FET: Forced expiratory technique
FEV_1_: Forced expiratory volume in 1 s
FVC: Forced vital capacity
HRQOL: Health related quality of life
O-PEP: Oscillating positive expiratory pressure
PEP: Positive expiratory pressure
